# Oral surgical procedures under local anaesthesia in day surgery

**DOI:** 10.1186/s12903-018-0648-6

**Published:** 2018-10-30

**Authors:** Abdulkadir Burak Çankaya, Çağrı Akçay, Neşe Kahraman, Banu Gürkan Köseoğlu

**Affiliations:** Faculty of Dentistry, Oral and Maxillofacial Surgery, İstanbul Üniversitesi, Istanbul, Turkey

**Keywords:** Day case surgery, Local anaesthesia, Gender, Type of treatment

## Abstract

**Background:**

The objective of this study was to analyze gender-stratified data of patients who underwent day surgery in a hospital based on the type of treatment, type of local anaesthesia, and local anaesthesia complications. By learning all these parameters, it is our main goal to find answers to questions such as what we can do in hospital conditions, what we can win, and what operations we can treat.

**Methods:**

A retrospective review was performed to assess hospital records of 10,750 dental patients who received oral surgery under local anaesthesia at the Istanbul University Department of Oral and Maxillofacial Surgery from August 2013 through June 2016.

**Results:**

Patients mostly received surgery for wisdom teeth, dental implants, or odontogenic cysts or tumours. Men aged 31–40 years (23.66%) and women aged 21–30 years (30.73%) were the largest groups undergoing operations. Surgery for an impacted tooth was the most common ambulatory procedure, accounting for 54.2% of operations. The second most common ambulatory procedure was dental implant surgery (10.2%), followed by root (7.4%), odontogenic cyst (7.2%), and impacted canine surgeries (6.4%). The most common age group receiving surgery was 21–30 years old (3304 patients, 60.75%). Twice as many women as men underwent surgery.

**Conclusions:**

Day case surgery is an expanding area of health care and a valuable method of treating patients in many aspects of oral surgical practice. Different medical and dental specialties can benefit from this ambulatory approach to treatment, which also reduces treatment costs.

## Background

Local anesthesia results from the prevention of the spread of the action potential so that the sensation cannot be transferred from the stimulus source, such as periodontium and tooth, to the brain. Sodium ions are necessary for a potential action to occur in associated nerve membrane. Local anesthetics work by blocking the entry of sodium ions into their channels. As a result, temporary increase in the permeability of the nerve membrane is prevented [1].

In 1996, Marin described the ambulatory surgery term as an optimal organizational model for multidisciplinary surgical care that permits effective and safe treatment of some patients without having to resort to conventional hospital beds. This concept involves interventions of moderate complexity, performed under general, local or regional anesthesia, which do not require hospitalization after surgery [2].

Day surgery is an expanding field of medical care and an invaluable method for treating patients in many aspects of oral surgery practice. Several medical and dental specialties can benefit from this outpatient treatment approach. In oral surgery, the accepted daily procedure includes tooth extraction, root fragment extraction, orthodontic dental fenestration, removal of small cystic lesions, hard and soft tissue biopsies and frenectomy, extraction of osteosynthesis plaques and wires from the jaws, soft tissue surgery, laser surgery and cryosurgery [3].

A retrospective study was performed to analyze medical records of patients who had been treated at the Istanbul University Department of Maxillofacial and Oral Surgery during the period from January 2010 to July 2013. The aim of this study was to gain insight into the types and frequencies of diagnoses and ambulatory surgical treatments at the Department of Oral Surgery in Istanbul University. We also explored whether there was a significant difference in procedures according to sex, age, and operation side. By learning all these parameters, it is our main goal to find answers to questions such as what we can do in hospital conditions, what we can win, and what operations we can treat.

## Methods

A total of 10,750 patients admitted to the Istanbul University Department of Oral Surgery from 2010 January to 2013 July were included in the study. The data were collected retrospectively from ambulatory outpatient operation protocols. The data was collected from clinic protocol records. The form for the ambulatory clinic protocols classified the patients according to date of arrival, age, sex, place of residence, and surgical treatments. After data collection, surgical treatments were categorized to facilitate statistical analysis.

Statistical calculations were performed with Number Cruncher Statistical System 2007 Statistical Software (Utah, USA) for Windows. Besides standard descriptive statistical calculations (mean and standard deviation, frequency, percentage), one-way ANOVA was used to compare multiple groups, the post hoc Tukey’s multiple-comparison test was used to compare subgroups, unpaired t-tests were used to compare two groups, and the Chi square test was performed to evaluate qualitative data. Statistical significance was established at *p* < 0.05.

## Results

A total of 10,750 dental patients were evaluated. This study included 4126 (38.38%) men and 6624 (61.62%) women, whose mean age was 37.70 years. Men aged 31–40 years (23.66%) and women aged 21–30 years (30.73%) were most common in this data set (Table 1).

**Table 1:** Total patient and gender distribution

	N	Mean	SD	Min	Max
Men	4126	38.17	13.47	8	90
Women	6624	37.41	13.32	7	86
Total	10,750	37.70	13.39	7	90

The 21–30-year age group was the largest, with 3304 patients (60.75%). The patient number in the group aged < 10 years was low because paediatric surgery generally employs general anaesthesia (Table 2). For both men and women, the most common surgery was for an impacted third molar (49.93 and 56.79%, respectively). On the other hand, cyst operations were more prevalent in men (10.18%) than in women (5.39%) (Table 3).

**Table 2:** Age distribution

Age (years)	Men (*n*)	Men (%)	Women (*n*)	Women (%)
< 10	5	0.12	6	0.09
11–20	301	7.30	525	7.93
21–30	1189	28.82	2115	31.93
31–40	1010	24.48	1533	23.14
41–50	863	20.92	1325	20.0
51–60	472	11.44	691	10.43
61–70	214	5.19	330	4.98
> 71	72	1.75	99	1.49

**Table 3:** Operation types in genders

Operation type	Men (*n*)	Men (%)	Women (*n*)	Women (%)	*p*
Impacted third molar	2060	49.93	3762	56.79	**0.0001**
Implant	429	10.40	669	10.10	0.62
Root	352	8.53	445	6.72	**0.0001**
Odontogenic cyst	420	10.18	357	5.39	**0.0001**
Impacted canine	230	5.57	454	6.85	0.008
Apical resection	239	5.79	333	5.03	0.086
Alveoloplasty	112	2.71	134	2.02	0.02
Vestibuloplasty	83	2.01	156	2.36	0.24
Odontogenic tumour	38	0.92	62	0.94	0.937
Frenectomy	37	0.90	44	0.66	0.175
Impacted premolar	30	0.73	42	0.63	0.565

This study included surgical treatments in the ambulatory clinic and ambulatory operation room. Impacted tooth operations were the most common ambulatory procedure at 54.2%. The second most common ambulatory procedure was dental implantation (10.2%), followed by root (7.4%), odontogenic cyst (7.2%), and impacted canine operations (6.4%). (Table 4).

**Table 4:** Operation types

Operation type	Number	%
Impacted third molar	5822	54.2
Implant	1098	10.2
Root	797	7.4
Odontogenic cyst	777	7.2
Impacted canine	684	6.4
Apical resection	572	5.3
Alveoloplasty	246	2.3
Vestibuloplasty	239	2.2
Odontogenic tumour	100	0.9
Frenectomy	81	0.8

The third molar was the most common impacted tooth, with a rate of 54.2%, followed by canines (6.4%) and premolars (0.7%) (Table 5).

**Table 5:** Impacted teeth numbers

Impacted teeth	Number	%
Third molar	5822	54.2
Canine	684	6.4
Premolar	72	0.7

Operations most commonly involved the mandibular arch (63.16%) (Table 6).

**Table 6:** Operated jaw numbers

Operated jaw	Number	%
Maxilla	3676	34.20
Mandible	6790	63.16
Maxilla and mandible	250	2.33
Temporomandibular joint (TMJ)	34	0.32

The posterior region was the most common surgical region for both mandible and maxilla (75.92%); posterior surgeries were nearly four times as common as those in the anterior region (22.49%) (Table 7).

**Table 7:** Operation region numbers

Operation region	Number	%
Posterior	8161	75.92
Anterior	2418	22.49
Posterior and anterior	137	1.27
TMJ	34	0.32

## Discussion

The potential advantages of day surgery include:

1. Patient benefits: Day surgery results in reduced waiting time and shortened hospital stay, leading to reduced risk of nosocomial infections, less interference with daily life activities, and the possibility of choosing from among various treatment alternatives.
2. Reduced costs: Day case surgery is more cost-effective than surgery with patient hospitalization. Savings in services are obtained, although the actual surgical costs are very similar in both modalities.
3. Improved quality patient care: Day case surgery can be performed with the same or even greater health care quality than surgery involving patient hospitalization. Previous studies showed that day surgery leads to better results than surgery with hospitalization, with fewer and less serious postoperative complications and a lower readmission rate.
4. Shortened waiting lists: The need for waiting lists is a complex phenomenon that cannot always be solved by increasing available health care resources [3].

Records of 10,750 patients treated in the oral ambulatory operation room were examined in this study. Surgically treated patients in a study by Cabov et al. had an age range of 5–88 years, with a median age of 37 years for men and 31 for women. The numbers in the present study did not differ significantly from other reports. Our patients had a similar age range, although women had a mean age of 37.

Syed et al. showed that in both males and females, impacted third molars were more prevalent in the mandibular arch than in the maxilla (49.5% vs. 48.62%). In that study, the male-to-female ratio for impacted third molars was 604: 109 (5.54: 1; *p* = 0.707). In our study, this surgery was also more common in the mandibular arch (63.16%), and the male-to-female ratio for the impacted third molar operation was 2060: 3762 (49.93, 56.79; *p* = 0.0001). According to Syed et al., the 20–25-year age group showed the highest prevalence of third molar impaction (64.5%), and the frequency decreased with increasing age. Similarly, in our study, the 21–30-year age group most commonly underwent this procedure, with 3304 patients (60.75%). In other studies, as in ours, the prevalence of impacted third molars was much higher in the mandibular than in the maxillary arch (49.4% vs. 18.4%) [4].

In the day surgery, the most common oral surgery routine is the extraction of the third molar. In a literature review performed by Ruiz-Mirete and Gay-Escoda, the lower thirds of the mandible were the most commonly impacted teeth [3]. Surgical removal of impacted teeth is most common procedure for several reasons:

1. Reducing in the caries index implies fewer extractions of non-impacted teeth. This may partly explain the greater extraction of impacted teeth, including third molars.
2. The increase in demand for orthodontic treatment has, in turn, increased the number of impacted teeth operations (extraction or fenestration of permanent upper canines) or the extraction of other permanent teeth, such as premolars.
3. The number of panoramic radiography devices have increased in the last 25 years. This additional radiographic technique allows for early detection of impacted teeth as well as control of eruption turnover and identification of dental and/or maxillary pathology requiring surgery [3].

In our study, more women than men underwent surgery, in accordance with previous findings. This could reflect increased prevalence of caries among women. In addition, this may be related to women’s increased interest in aesthetics and health care. Women and men had more operations for impacted wisdom tooth and implants in other countries A study in North India also found that women had more surgeries for impacted teeth more than men [5]. In our study, the number of operations for impacted wisdom teeth was 2060 (49.93%) in men and 3762 (56.79%) in women In Ontario, Canada, the most common day surgery procedure was extractions. In the Dental Clinic of the University of Barcelona, 15.34% of oral surgery treatments were performed on patients younger than 18 years of age. In our study, the number of young patients was significantly lower (7.79%) because most younger patients were scheduled for surgery under general anaesthesia and were therefore not included in this survey. General anaesthesia represents a risk factor for bacteremia following dental exrtractions (BDE), increasing its prevalence and duration [6]. On the other hand, patients subjected to ambulatory surgery should have a very low risk of serious complications, as they are selected according to their general condition (i.e., the absence of serious systemic diseases) [3].

## Conclusions

Day surgery can be adapted to almost all the oral surgery interventions because these procedures are short-term operations that can be performed with regional anesthesia. Numerous factors contribute to the growing popularity of outpatient surgery;

- Surgery with admission of patients to the hospital costs very much;
- Recent developments at technological advances have led to many outpatient operations;
- The new drugs used under anesthesia allow sedation or general anesthesia with rapid recovery and without side effects;
- Day surgery influences less the daily life of patient.

## Abbreviation

BDE: Bacteremia following dental extractions
